# Embelin-induced apoptosis of HepG2 human hepatocellular carcinoma cells and blockade of HepG2 cells in the G2/M phase via the mitochondrial pathway

**DOI:** 10.3892/etm.2012.637

**Published:** 2012-07-11

**Authors:** ASAF TAGHIYEV, DEGUANG SUN, ZHEN MING GAO, RUI LIANG, LIMING WANG

**Affiliations:** Department of General Surgery, The Second Affiliated Hospital of Dalian Medical University, Dalian 116027, P.R. China

**Keywords:** embelin, XIAP, mitochondria, apoptosis, human hepatocellular carcinoma HepG2 cells

## Abstract

Embelin is a small-molecule inhibitor extracted from plants of the Myrsinaceae family demonstrating specific inhibition of the X-linked inhibitor of apoptosis protein (XIAP) to affect the proliferation and apoptosis of various types of tumor cells. However, the mechanism of action for this effect remains unclear. The purpose of the present study was to investigate the role of the mitochondrial pathway in embelin-induced HepG2 human hepatocellular carcinoma cell apoptosis and the effect of embelin on the cell cycle. HepG2 human hepatocellular carcinoma cells were treated with different doses of embelin. The MTT method was used to determine cell viability, and flow cytometry was used to assess the rate of apoptosis and the changes in mitochondrial membrane potential; the cell cycle was also analyzed. Western blot analysis was performed to determine the expression levels of the apoptosis-associated proteins Bax, Bcl-2 and the caspase family. The results revealed that embelin induced the apoptosis of the HepG2 cells in a dose- and time-dependent manner. In addition, embelin caused changes in mitochondrial membrane potential. Flow cytometric analysis demonstrated that embelin caused blockade of the HepG2 cells in the G2/M phase of the cell cycle.

## Introduction

The incidence of hepatocellular cancer (HCC) ranks fifth among all malignant tumors worldwide. Moreover, HCC is the third most common cause of cancer-related mortality worldwide (1–4), with more than 600,000 individuals succumbing to the disease each year. Similar to other solid tumors, surgical resection is the main treatment option for HCC (5), yet the high frequency of local recurrence and distant metastasis remain the largest obstacles to the survival of post-operative HCC patients (6). Due to the difficulty in the early diagnosis of HCC, most patients are treated during advanced or late-stage disease, and only a small percentage of patients have the chance of radical treatment. Therefore, the development of new approaches for clinical therapy of liver cancer is of utmost importance.

Embelin is a small-molecule inhibitor extracted from plants of the Myrsinaceae family. It is a polyphenolic compound that inhibits the X-linked inhibitor of apoptosis protein (XIAP) through bonding with the Smac bonding site in the BIR3 domain in the XIAP protein molecule (7,8). Previous studies have demonstrated that embelin has anti-inflammatory and anti-oxidative effects (9–11). Embelin has been found to suppress the growth of various types of tumor cells including breast, colon, prostate and pancreatic cancer cells. However, there have been no reports on whether embelin inhibits the growth of human hepatocellular carcinoma cells. Moreover, the detailed mechanism of embelin against tumors remains unknown.

Studies have demonstrated that apoptosis of liver cancer cells is closely associated with the mitochondrial pathway (16–18). Our previous study found that embelin promoted changes in the expression of the Bax and Bcl-2 proteins. The migration of Bax and Bcl-2 proteins resulted in changes in mitochondrial membrane potential, which in turn released cytochrome *c* and activated the caspase family, resulting in apoptosis (19). A previous study also demonstrated that the Bax and Bcl-2 proteins are two important targets of anticancer drugs (20).

The present study was designed to investigate the impacts of embelin *in vitro* on the apoptosis of HepG2 human hepatocellular carcinoma cells and the cell cycle, and to explore the embelin-induced apoptosis signaling pathway. We found that via the regulation of the Bax and Bcl-2 proteins, embelin releases cytochrome *c* and activates the caspase family to induce the apoptosis of HepG2 cells.

## Materials and methods

### Materials and chemicals

Embelin (≥99% purity), propidium iodide (PI), JC-1 and MTT were purchased from Sigma Inc. (St. Louis, MO, USA). The Annexin V-FITC/PI kit was purchased from BD Biosciences (Franklin Lakes, NJ, USA). All antibodies were from Santa Cruz (Santa Cruz, CA, USA).

### Cell culture

HepG2 human hepatocellular carcinoma cells were from American Type Culture Collection (ATCC). Cells after cell passage were inoculated in RPMI-1640 culture medium (Gibco-BRL, NY, USA) containing 10% fetal calf serum (Hyclone Laboratories, UT, USA), 100 U/ml penicillin and 100 U/ml streptomycin. The cells were then cultured in an incubator containing 5% CO_2_ and 95% oxygen at 37°C.

### Cell viability

HepG2 cells in logarithmic growth phase were inoculated in a 96-well culture plate with a density of 1×10^5^ cells/ml. After the cells grew to adherence, different doses of embelin were administered to various groups with 6 duplicate wells for each concentration. TAa negative control group without embelin treatment was established. All the cells were placed in a 5% CO_2_ incubator for further culture for 24, 48 and 72 h prior to color reaction. Each well was supplemented with 20 μl of MTT (5 mg/ml) and cultured in a CO_2_ incubator for 4 h before the culture solution was disposed of. DMSO (150 μl)was added to each well at room temperature and oscillation was carried out for 10 min and the OD values (A570nm) of each well were measured with a microplate reader.

### Evaluation of apoptosis

During the early stage of apoptosis, phosphatidylserine was exposed outside the membrane from the interior of the cell membrane due to the lack of symmetry of the cell membrane. Trypsin (0.25%) was digested to collect the cells of all groups, and the cell density was adjusted to 1×10^6^ cells/ml. Annexin V-FITC (5 μl), and 5 μl of PI were added, respectively, for dyeing (30 min at 4°C) before analysis with flow cytometry.

### Analysis of the cell cycle by flow cytometry

The cells of all groups were collected and the trypsin method was used. The cells were washed with PBS 3 times and fixed at 4°C with 75% cold ethanol overnight. The ethanol was removed and the cells were washed in PBS 3 times. The cell density was adjusted to 1×10^6^ cells/ml with a final volume of 100 μl. DNAStain comprehensive dye liquor (500 μl) with RNase at a final concentration of 50 mg/l, PI at a final concentration of 100 mg/l and Triton X-100 at a final concentration of 1 ml/l was added and cells were stored at room temperature in a dark place for 30 min before analysis with flow cytometry.

### Flow cytometric analysis of mitochondrial membrane potential

The cell density was adjusted to 1×10^6^ cells/ml, JC-1 dye liquor was added with a final concentration of 10 μg/ml and even mixing was carried out; the cells were cultured for 30 min at 37°C in a dark place. Analysis with flow cytometry was performed (BD Biosciences). JC-1 monomer and polymer fluorescent signals from FL1 and FL2 probes, respectively, were obtained. FL1-H indicated green fluorescence intensity and FL2-H indicated red fluorescence intensity. Quantitative analysis was carried out with Cellquest analysis software.

### Western blot assay

The cells of all groups were prepared using the trypsin method and 2 ml of lysis solution (50 mM of Tris-HCl, 137 mM of Nacl, 10% glycerin, 100 mM of sodium vanadate, 1 mM of PMSF, 10 mg/ml of apotinin, 10 mg/ml of eupeptin, 1% NP-40 and 5 mM of cocktail; pH 7.4) was added for cell lysis to retrieve the proteins. Proteins were loaded following the BCA method of rationing. The proteins were separated with SDS-PAGE. The proteins were shifted to PVDF membranes using a semidrying method and sealed with 5% skim mild powder at 4°C overnight. The membranes were washed with TBST and the first antibody was added at 37°C for hybrid for 1 h before bleaching with TBST. The second antibody was added at 37°C for hybridization for 1 h before bleaching with TBST and completing color reaction for 5 min with autoradiography. Quantity One was used for optical density value analysis and measurement. The results are presented as optical density value/actin optical density value of the samples.

### Statistical analysis

SPSS 16.0 statistical software was used for statistical analysis. Values are shown as the mean ± SD. Statistical analysis was carried out using the Student’s t-test. Differences between groups were considered statistically significant at p<0.05.

## Results

### Embelin-induced inhibition of HepG2 human hepatocellular carcinoma cell growth

**Figure 1:**
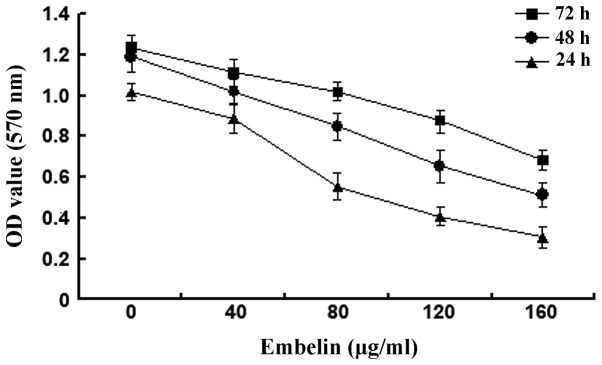
Inhibition of HepG2 human hepatocellular carcinoma cell growth by embelin. Cell viability was assessed using the MTT method after HepG2 cells were treated with different concentrations of embelin (0, 40, 80, 120 and 160 μg/ml) for 24, 48 and 72 h. The results are representative of six independent experiments.

Embelin of different concentrations (0, 40, 80, 120 and 160 μg/ml) was administered to HepG2 cells for 24, 48 and 72 h before the MTT method was used to determine the cell viability (Fig. 1). The results showed that A570 values of HepG2 cells gradually decreased when the concentration of embelin increased from 40 to 160 μg/ml and the A570 value decreased the most when the concentration of embelin was at 120 μg/ml. This suggests that embelin has potent inhibitory effects on the growth of HepG2 cells.

### Embelin-induced apoptosis of HepG2 human hepatocellular carcinoma cells

**Figure 2:**
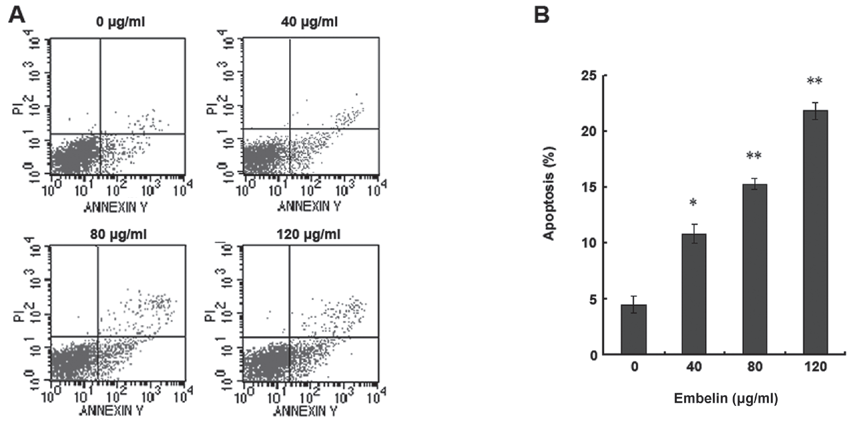
Embelin-induced apoptosis of HepG2 human hepatocellular carcinoma cells. (A) Annexin V/PI dye was used to determine the rate of apoptosis after HepG2 cells were treated with different doses of embelin (0, 40, 80 and 120 μg/ml) for 48 h. (B) The histogram shows the HepG2 apoptosis rate (%).^*^P<0.05, ^**^P<0.01. The data are representative of three independent experiments

HepG2 human hepatocellular carcinoma cells were treated with different doses of embelin (0, 40, 80 and 120 μg/ml) for 48 h and flow cytometry was used to assess the rate of apoptosis (Fig. 2). Our results indicate that with the increase of the embelin dose, the quantity of HepG2 apoptosis rose significantly (Fig. 2A and B). Therefore, embelin induces apoptosis of HepG2 human hepatocellular carcinoma cells in a dose-dependent manner.

### Embelin induces apoptosis of HepG2 human hepatocellular carcinoma cells through the mitochondrial pathway

**Figure 3:**
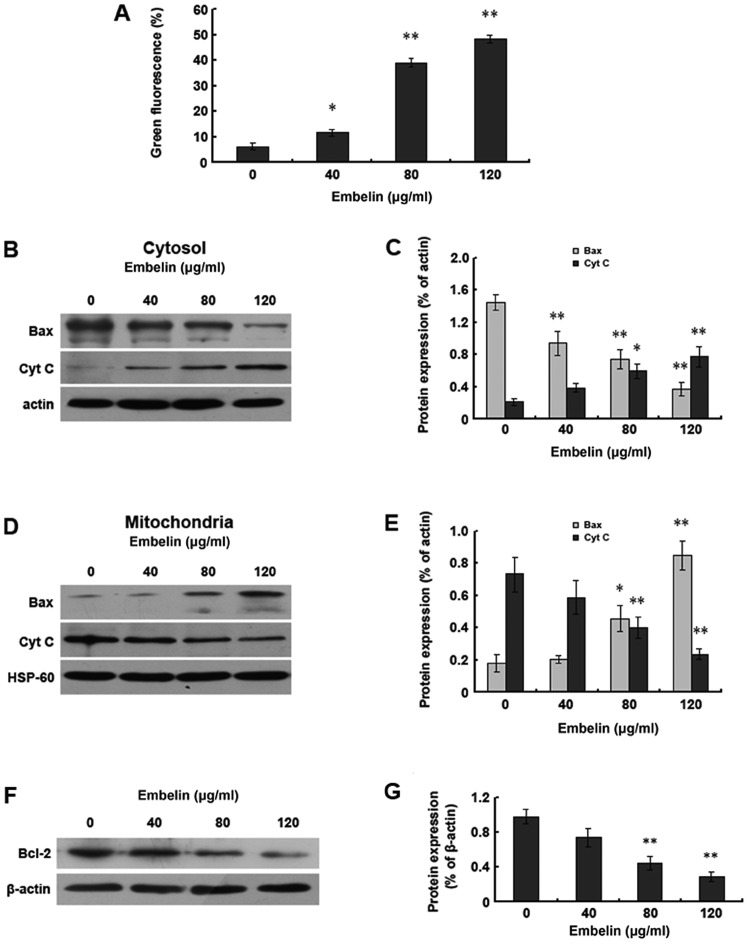
Induction of HepG2 human hepatocellular carcinoma cell apoptosis by embelin via the mitochondrial pathway. After HepG2 cells were treated with different doses of embelin (0, 40, 80 and 120 μg/ml) for 48 h, (A) JC-1 dye flow cytometry was used to analyze the changes in mitochondrial membrane potential; (B and D) western blot analysis was used to analyze the expression levels of the Bax and cytochrome *c* proteins inside the cytoplasm and mitochondria. (F) Western blot analysis was used to analyze Bcl-2 protein expression levels. (C, E and G) The western blotting results were further analyzed using Gel-Pro Analyzer 4.0 software. ^*^P<0.05, ^**^P<0.01. Results are representative of three independent experiments.

In order to prove the relationship between the induction of HepG2 cell apoptosis by embelin and the mitochondrial pathway, JC-1 coloration was used to assess the changes in the mitochondrial membrane potential. The results demonstrated the gradual decrease of the mitochondrial membrane potential along with the increase of the dosage of embelin, with the change of the mitochondrial membrane potential occurring before apoptosis (Fig. 3A). Bax gene shift and the release of cytochrome *c* are another sign of apoptosis. We observed the levels of Bax and cytochrome *c* inside the cytoplasm and mitochondria using western blot analysis and found that with the increase of the embelin dosage, the Bax protein levels inside the cytoplasm decreased whereas the cytochrome *c* levels increased. However, the levels of Bax and cytochrome *c* inside the mitochondria were just opposite to those inside the cytoplasm (Fig. 3B–E). We also found that the levels of the Bcl-2 protein decreased with the increase of the embelin dosage (Fig. 3F and G). Our data demonstrate that embelin is able to change the mitochondrial membrane potential to promote the shift of Bax and Bcl-2 as well as the release of cytochrome *c,* which results in the apoptosis of HepG2 human hepatocellular carcinoma cells.

### Effect of embelin on the expression levels of human hepatocellular carcinoma HepG2 apoptosis-related proteins

**Figure 4:**
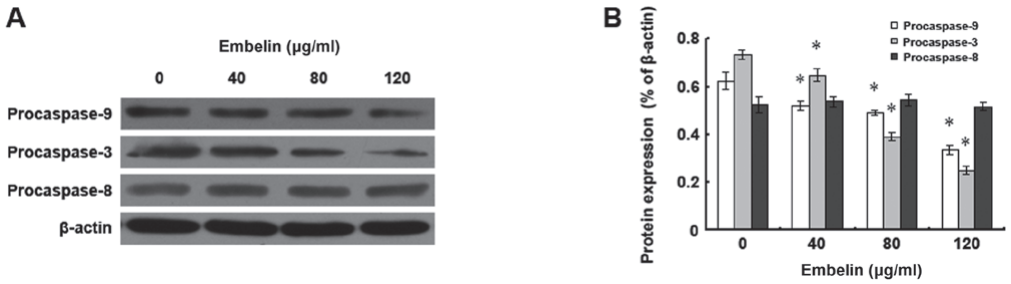
Effect of embelin on the expression levels of the HepG2 human hepatocellular carcinoma cell apoptosis-related proteins, caspase 3, 8 and 9. Forty-eight hours after the HepG2 cells were treated with 120 μg/ml of embelin, (A) western blot analysis was used to analyze the expression levels of the caspase 3, 8 and 9 proteins. (B) The western blotting results were further analyzed using Gel-Pro Analyzer 4.0 software. ^*^P<0.05. Results are representative of three independent experiments.

In order to study the impact of embelin on the expression levels of HepG2 human hepatocellular carcinoma cell apoptosis-related proteins, different doses of embelin were administered to HepG2 cells for 48 h, and western blot analysis was used to examine the expression of the procaspase 3, 8, 9 proteins (Fig. 4). It was found that after the treatment with 120 μg/ml of embelin, the expression levels of both the HepG2 cell procaspase 3 and 9 proteins decreased significantly, while there was no significant change in the expression level of the procaspase 8 protein. Our data demonstrate that the HepG2 embelin-induced apoptosis may be through the mitochondria-mediated caspase 3 and 9 pathways.

### Embelin-induced HepG2 human hepatocellular carcinoma cell cycle blockade in the G2/M phase

**Figure 5:**
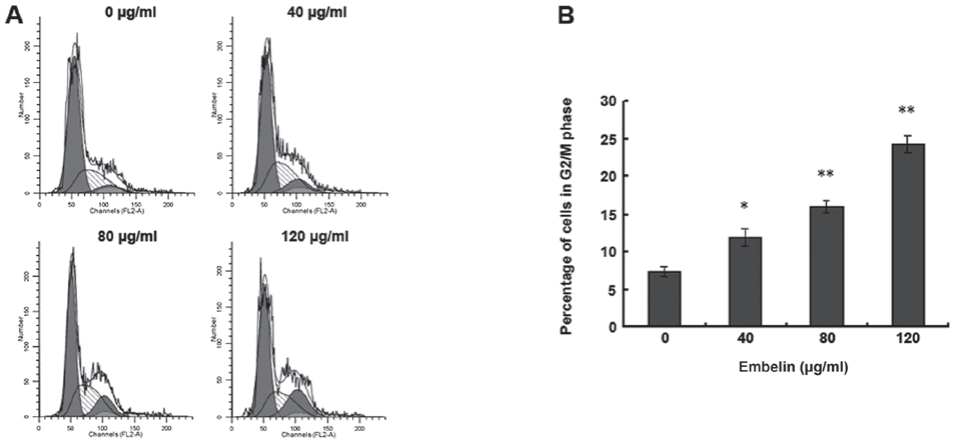
Effect of embelin on the HepG2 human hepatocellular carcinoma cell cycle. After different doses of embelin were administered to HepG2 cells for 48 h, (A) flow cytometry was used to analyze the cell cycle; (B) the histogram shows the HepG2 cell cycle (%). ^*^P<0.05, ^**^P<0.01. The data are representative of three independent experiments.

In order to investigate whether embelin effects the HepG2 human hepatocellular carcinoma cell cycle, we used flow cytometry for the analysis. The results showed that 48 h after administering different doses of embelin to HepG2 cells, the HepG2 cells were blocked in the G2/M phase, which was apparently higher than that of the control group (Fig. 5). This indicates that embelin is able to inhibit the G2/M phase percentage of HepG2 cells to restrain the hepatocellular carcinoma cell proliferation.

## Discussion

Kerr *et al* (21) were the first to report on the concept of apoptosis. Apoptosis is ubiquitous in most of the tumor cells and plays an important role in the genesis and progression of tumors (22,23). Previous studies have demonstrated that antitumor drugs generally inhibit tumors through the induction of apoptosis of sensitive tumor cells, and their antitumor effects relate to the interior activation of the apoptosis of the tumor cells induced by the drugs. Therefore, the induction of apoptosis to treat tumors has become a new target for the development of antitumor drugs and constitutes a new direction in the current research in tumor pharmacology.

Embelin is a small-molecule inhibitor exhibiting specific inhibition of XIAP that affects the proliferation and apoptosis of various tumor cells. Embelin has gained much worldwide attention for its antitumor effects. As shown in previous studies, embelin inhibits the proliferation of various tumor cells, with particularly significant effects on breast and pancreatic cancer as well as on other solid tumor cells (24,15). Our results support the hypothesis that embelin induces the apoptosis of human hepatocellular carcinoma cells. Flow cytometry revealed that after human hepatocellular carcinoma cells were treated with different doses of emblin for 48 h, the Annexin V-positive rate of the cells increased in a dose-dependent manner, indicating that embelin can induce human hepatocellular carcinoma apoptosis instead of cell death. Embelin is also capable of restraining the cell cycle alterations of hepatocellular carcinoma cells to cause blockade of the cell cycle in the G2/M phase so as to change the cell cycle progress to induce apoptosis.

There are two main signaling pathways to trigger apoptosis: the endogenous mitochondrial pathway and the exogenous death receptor pathway (25). Our research found that after human hepatocellular carcinoma cells were treated with different doses of emblin for 48 h, Bax and Bcl-2 migrated, the mitochondrial membrane potential decreased, and cytochrome *c* was released. These findings indicate that the induction of human hepatocellular carcinoma cell apoptosis by embelin is mediated by the mitochondrial pathway. The Bcl-2/Bax family is the key factor in regulating the endogenous mitochondrial apoptosis pathway (26,27). With the pro-apoptosis effect, the Bax gene migrates from the cytoplasm to the mitochondrial outer membrane, changing the permeability of the mitochondrial outer membrane to promote the release of cytochrome *c* from the mitochondria (28). Moreover, the Bcl-2 protein can regulate the opening and closing of mPTP. mPTP, a non-selective pathway spanning over the inner and outer mitochondrial membrane and the mitochondrial irritation receptor, is considered to trigger the life or death of the cells (29). Our study found that with embelin treatment, the Bcl-2 protein level inside the cytoplasm decreased gradually while the release of cytochrome *c* increased step by step. This indicates that embelin induces the activity of Bcl-2, makes mPTP open irreversibly, causes further changes in the permeability of the mitochondrial membrane and promotes the release of cytochrome *c*. The combination of these two factors results in apoptosis.

It is well known that the caspase family activates apoptosis-related protease when apoptosis occurs (30), so the activation of the caspase family is an important prerequisite for apoptosis. We analyzed the changes in the procaspase 9, 8, 3 proteins using western blot analysis after the treatment with embelin and found that the expression levels of procaspase 9 and 3 decreased significantly when hepatocellular carcinoma apoptosis occurred, but no apparent changes in procaspase 8 expression were observed. The release of cytochrome *c* from mitochondria to activate caspase 9, 3 is a key step of the apoptosis pathway (31). However, no apparent changes in procaspase 8 expression were found. These results indicate that the induction of hepatocellular carcinoma apoptosis by embelin is realized via the endogenous mitochondrial pathway instead of the exogenous death receptor pathway.

In summary, our study demonstrated that embelin releases cytochrome *c* and activates the caspase family, resulting in the induction of hepatocellular carcinoma apoptosis by regulating the action of the Bcl-2/Bax family on the mitochondrial pathway. Embelin may offer important contributions in the development of a new drug to prevent and cure HCC in the future.
